# Reduced hospitalization rates are not associated with increased mortality or readmission rates in an emergency department in Israel

**DOI:** 10.1186/s13584-018-0265-5

**Published:** 2018-11-20

**Authors:** Sharon A. Greenberg, Pinchas Halpern, Tomer Ziv-Baran, Ronni Gamzu

**Affiliations:** 1 Emergency medicine department, Tel-Aviv Sourasky Medical Center, 6 Weizmann st, 6423906 Tel-Aviv, Israel; 2 Department of Epidemiology and preventive Medicine, Sackler Faculty of Medicine, Tel Aviv University, Tel-Aviv, Israel; 3 Managment, The Tel Aviv Sourasky Medical Center, Tel Aviv, Israel

## Abstract

**Background and Aim:**

In 2011 the Israeli Ministry of Health (MOH) instructed hospitals to limit occupancy in the internal medicine wards to 120%, which was followed by a nationwide reduction in hospitalization rates. We examined how readmission and mortality rates changed in the five years following the changes in occupancy rates and hospitalization rates.

**Methods:**

All visits to the Tel Aviv Medical Center internal Emergency Medicine Department (ED) in 2010, 2014 and 2016 were captured, with exclusion of visits by patients below 16 of age and patients with incomplete or faulty data. The main outcomes were one-week readmission rates and one-month death rates. The secondary outcomes were admission rate, ED visit length & admission-delay time (minutes), and rates of admission-delayed patients.

**Results:**

After exclusion, a total of 168,891 internal medicine ED patients were included in the analysis. Mean age was 58.0 and 49% were males. During the relevant period (2010–2016), total medical ED visits increased by 11% - 53,327, 56,588 and 59,066 in 2010, 2014 and 2016 respectively. Hospitalization rates decreased from 46% in 2010 to 35% in 2015 (*p* < 0.001), with the most prominent reduction in the elderly population. One-week readmission rates were 6.5, 6.4 and 6.7% in 2010, 2014 and 2016 respectively (*p* = 0.347 and *p* = 0.21). One-month mortality was similar in 2010 and 2014 (4.4 and 4.5%, *p* = 0.388) and lower in 2016 (4.1%, *p* = 0.048 compared with 2010). Average ED visit length increased from 184 min in 2010 to 238 and 262 min in 2014 & 2016 (*p* < 0.001 for both) and average delay time to ward admission increased from 97 min in 2010 to 179 and 240 in 2014 & 2016 (*p* < 0.001 for both). In 2010 24% of the admitted patients were delayed in the ED more than 2 h, numbers that increased to 53% in 2014 and 66% in 2016 (*p* < 0.001 for both).

**Conclusion:**

Following the 2011 MOH’s decision to establish a 120% occupancy limit for internal medicine wards along with natural growth in population volume, significant changes were noted in the work of a large, presumably representative emergency department in Israel. Although a steady increase in total ED visits along with a steady reduction in hospitalization rates were observed, the readmission and mortality rates remained low. The increase in the average length of ED visits and in the delay from ED admission to a ward reflects higher burden on the ED.

The study was not able to establish a causal connection between the MOH directive and the subsequent changes in ED activity. Nonetheless, the study has significant potential implications for policy makers, including the presence of senior ED physicians during afterhours, creation of short-stay diagnostic units and proper adjustments in ED size and personnel.

## Background

Following the 2011 Israeli Ministry of Health instruction (MOH) to limit internal medicine wards occupancy rates to 120% of official ward capacity [1], a marked reduction was noticed in hospital admission rates from the emergency department (ED) to the internal medicine departments. This reduction was prominent within the first year of policy change (55.6 admissions per 1000 capita and 50.0 per 1000 capita in 2010 and 2012 respectively), with a smaller decrease in the later years (49.3 per 1000 in 2015), and is a continuing trend [2]. In the Israeli Ministry of Health document from 2017, Haklay et al. describe at the national level, that while a 9% increase in internal medicine departments admission rates was documented between 2000 to 2010, this tendency switched to a 4% decrease in admission rates between 2010 to 2014 [3]. However, ED discharge and readmission rates were not addressed in these studies, despite the concern that undesired side effects of a rise in discharge emergency ED rates might include a rise in readmissions rates, as well as a decline in the quality of care in the ED.

In previous studies, readmission rates measured at 30 days varied between 10 to 12% for general medical population, and 15–17% for elderly patients [4–6], while one-week readmission rates were 8–14% [7]. Rates for a short-term return within 72 h following ED discharge (“bounce-back” phenomenon) approximated 3%, ranging from 0.5 to 5.5% [8–10]. As for mortality, increased mortality rates in over-crowded hospitals were reported in Australia and Denmark [11, 12], and in a recent cohort study Obermeyer et al. reported increased early death rates among hospitals with lower admission rates [13].

It is important to mention that greater ED crowding was not associated with increased readmission rates [14]; and that greater hospital resources (i.e. more beds) were associated with increased hospitalization rates, but with no mortality benefit [15].

## Aim

As a tertiary medical center treating more than 50,000 internal medicine ED patients per year, our purpose was to study the influence of changes in ED discharge rates on readmission and death rates. The importance of this study is to understand the effect of reduced admission rates on public health.

## Methods

### Population and main parameters

The Tel-Aviv Sourasky Medical Center (TASMC) ED is a Level 1 adult Trauma ED, with a total of 135,238 medical-surgical visits in 2010 and 146,753 in 2016. The ED is divided into internal and surgical wings. We analyzed data of all patients admitted to the internal medicine ED at the TASMC in the years 2010, 2014 and 2016. We excluded patients who were (1) age below 16; (2) had incomplete data; we further excluded cases of ED stays > 24 h.

We collected age, ED visit time, admission rates, delay time in ED (after patient was signed for hospitalization by ED physician), readmission rates at one week, and death within one month of ED visit. General population data was obtained from the Israeli central bureau of statics.

### Statistical analysis

Categorical variables were reported as frequencies and percentages and continuous variables were reported as means and standard deviations (SD) or medians and interquartile ranges (IQR). Continuous variables were evaluated for normal distribution using histograms and Q-Q Plots. Generalized linear models were used for the univariate and multivariate analysis. The different means were compared by Student t test for normally distributed variables and by the Mann–Whitney U test for non-normally distributed variables. To assess associations among categorical variables, we used the χ2 test. A two-tailed *p* < 0.05 was considered statistically significant. Analyses were performed with IBM SPSS Statistics for Windows, Version 22.0 (IBM Corp., Armonk, NY, USA).

## Results

**Fig. 1:**
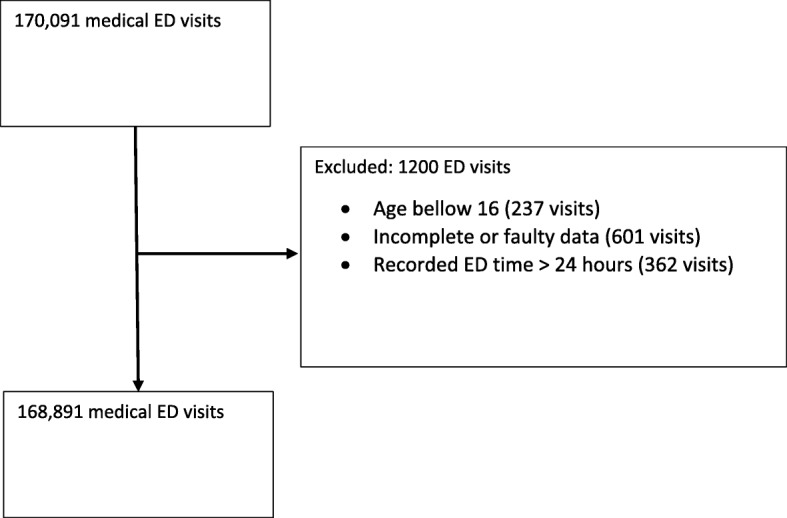
Study Population

A total of 170,091 visits were recorded in the selected years. After exclusion, a total of 168,891 visits were analyzed. See Fig. 1.

The mean age of the entire cohort of patients was 58.0 years, 49.2% males; 67,347 (39.9%) were admitted to an inpatient ward. ED readmission rates at one week were 6.6% (*n* = 11,068). One-month death rates were 4.3% (*n* = 7299). Average length of stay (LOS) in the ED was 229.1 ± 170.6. Admission delay time (calculated for hospitalized patients as time between recorded decision of hospitalization and actual time of admission to the inpatient ward) was 176.8 ± 185.9 min. See Table 1 for complete data.

**Table 1:** Cohort data and years comparison

Variable	Entire cohort	2010	2014	2016	Sigma (2010–2014)	Sigma (2010–2016)	Sigma (2014–2016)
N	168,891	53,237	56,588	59,066			
Male, N (%)	83,177 (49.2)	26,030 (48.9)	27,970 (49.4)	29,177 (49.4)			
Age (years), Mean (st. dev)	58.03 (22.3)	57.77 (22.4)	58.13 (22.2)	58.18 (22.3)	0.008	0.002	0.674
Admitted, N (%)	67,347 (39.9)	24,727 (46.4)	21,880 (38.7)	20,740 (35.1)	0.000	0.000	0.000
Discharged, N (%)	101,544 (60.1)	28,510 (53.6)	34,708 (61.3)	38,326 (64.9)	0.000	0.000	0.000
ED time (minutes), Mean (st. dev)	229.1 (170.6)	183.6 (127.96)	237.5 (167.42)	262.0 (196.36)	0.000	0.000	0.000
Delay Time (minutes), Mean (st. dev)	167.7 (181.93)	97.25 (94.4)	178.8 (176.18)	239.5 (229.79)	0.008	0.000	0.000
One-month mortality, N (%)	7,299 (4.3)	2,326 (4.4)	2,533 (4.5)	2,440 (4.1)	0.388	0.048	0.004
Readmission 1 week, N (%)	11,068 (6.6)	3,479 (6.5)	3,619 (6.4)	3,970 (6.7)	0.347	0.21	0.025
Delay > 2 h, N (%)	31,299 (46.8)	5,856 (23.7)	11,679 (53.4)	13,764 (66.4)	0.000	0.000	0.000

Admission & discharge rates, total Emergency department (ED) visit length & delay time (minutes), one-month mortality and one-week readmission rates in the years 2010, 2014 & 2016 in the Tel Aviv Sourasky Medical Center (TASMC). Number of patients that are delayed in the ED more than 2 h after decision of hospitalization took place increased significantly

### Steady rise in total ED visits, steady reduction in admission rates

While the total number of ED visits increased steadily each year (+ 6.3% and + 4.4% increment between 2010 and 2014 and 2014–2016 respectively), the total number of admissions and admission rates decreased significantly (− 11.5% and − 5.2% difference between 2010 and 2014 and 2014–2016 respectively). The mean age was 57.7 years in 2010, 58.13 in 2014 and 58.18 in 2016 (*p* < 0.01).

In 2010, 24,727 of all the ED visits ended in hospitalizations, i.e. admission rate of 46.4%. In 2014 however, admission rates were much lower: 38.7% (*n* = 21,880, *p* < 0.001). This trend continued in 2016: only 20,740 of 59,066 visits ended in admission to a hospitalization ward, for an admission rate of 35.1% (*p* < 0.001 compared with either 2010 and 2014) – see Table 1.

**Fig. 2:**
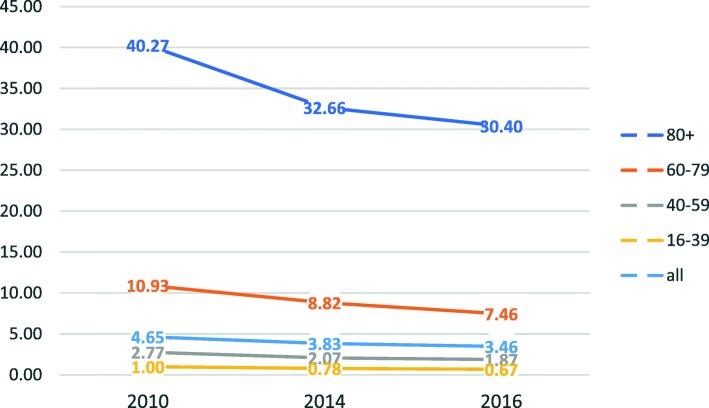
Admissions per 1000 capita dropped significantly, especially in the elderly

We further divided our cohort into four age categories – 16-39, 40–59, 60–79 and 80+. For each category, matching general population statistics were obtained from the national central bureau of statics. The adjusted admission rate per 1000 capita decreased in this period from 4.65 in 2010 to 3.83 in 2014 and 3.46 in 2016. The rate of hospitalization increased with age, while in each age category the rate of admission decreased with the years progression – see Table 2 and Fig. 2 (admissions per 1000 capita by age groups).

**Table 2:** Age adjusted admission rate

	2010				2014				2016			
age group	Total visits	Admitted	Admission rate	per 1000 capita	Total visits	Admitted	Admission rate	per 1000 capita	Total visits	Admitted	Admission rate	per 1000 capita
16–39	14607	2666	18.3%	0.99	14966	2183	14.6%	0.78	15641	1939	12.4%	0.67
40–59	11434	4381	38.3%	2.77	12196	3565	29.2%	2.07	12508	3326	26.6%	1.87
60–79	15661	9303	59.4%	10.93	17224	8459	49.1%	8.82	18033	8087	44.8%	7.46
80+	11535	8377	72.6%	40.27	12202	7673	62.9%	32.66	12884	7388	57.3%	30.40

Admission rates dropped in all age groups. This Change was profound in the elderly population, where age adjusted admissions per 1000 capita dropped by ¼

### Readmission & mortality rates remained low

Seven-day readmission rates remained unchanged. Rates were 6.5% in 2010 (*n* = 3479) and 6.4% in 2014 (*n* = 3619), *p* = 0.347. This trend remained unchanged in 2016, with readmission rates of 6.7% (*n* = 3970), *p* = 0.21 compared with 2010.

**Fig. 3:**
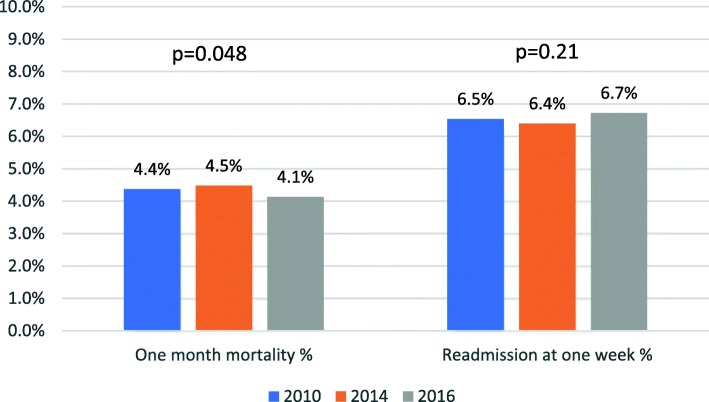
One-month mortality and one-week readmission rates remained low

Death rates at one month were also similar between the years, being 4.4% (*n* = 2326) in 2010 and 4.5% (*n* = 2533) at 2014, *p* = 0.388. In 2016 death rates were slightly lower – 4.1% (*p* = 0.048 and *p* = 0.004 compared with 2010 and 2014 respectively). See Fig. 3, Tables 1.

### Ed LOS and admission delay time are on the rise

**Fig. 4:**
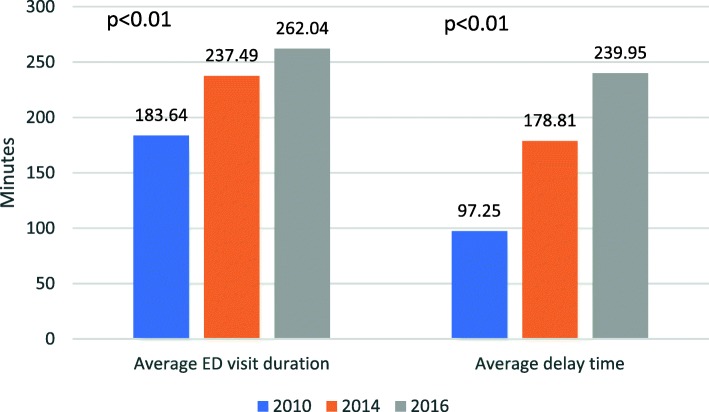
The length of visit in the Emergency department (ED) and delay time (after patient was signed for hospitalization by ED physician) (minutes) both increased significantly within the cohort period

In 2010, the average LOS of ED visits was 183.6 min compared with 237.5 in 2014 and 262.1 in 2016 (mean difference of 53.9 and 78.4 min respectively; 95% CI 52.1–55.6, *p* < 0.001 and 95% CI 76.4–80.4, *p* < 0.001 respectively). Among hospitalized patients, delay time between decision of hospitalization and actual time of admission to the inpatient ward grew from an average of 97.3 min in 2010 to 178.8 min in 2014 and 239.9 in 2016 (mean difference 81.6 and 142.7 min respectively; 95% CI 79.0–84.1; *p* < 0.001 and 95% CI 139.5–145.9, *p* < 0.001 respectively). See Fig. 4 and Table 1.

Of the 67,347 inpatient admissions of the cohort, 31,299 (46.8%) were delayed more than two hours in the ED, after decision of hospitalization took place. While in 2010 less than a quarter of the admitted patients were delayed more than 2 h (*n* = 5856, 23.7%), in 2014 more than half of the admissions were delayed more than 2 h (*n* = 11,679, 53.4%, *p* < 0.001). This tendency increased in 2016, as two thirds of the hospitalizations were delayed more than 2 h in the ED (*n* = 13,764, 66.4%, *p* < 0.001).

To complete this study, we examined data of bed occupancy in the internal medicine wards in the cohort time. The total bed occupancy in the internal medicine wards (measured at midnight every day) in the TASMC was 120,148, 119,380 and 114,909 in the years 2010, 2014 and 2016 respectively, which reflects a 4.4% decrease from 2010 to 2016.

## Discussion

The main findings in our study is that although admission rates declined by one fifth (from 46.4% to 38.7% in 2014 and 35.1% in 2016), one-week readmission and one-month mortality rates remained low and unchanged. Thus, discharge criteria from the ED seem to be appropriate and have not changed with increasing discharge rates. The increase in discharge rates is more profound in the elderly population – a one quarter reduction in hospitalization rates in the 60–79 and 80+ age groups (Fig. 2). Perhaps, prior to the 2011 change in policy, there were excess inpatient admissions which the pressure caused by the policy change reversed. At the same time, significant numbers of patients admitted after hours are now held in the ED overnight and are then discharged by senior ED staff the next morning (PH - personal communication), adding significantly to the decline in admission rates.

We noticed two trends concerning the characteristics of the population: between 2010 and 2014 the number of total visits increased by 6.3% and the population grew 4.3 months older. In 2016 we recorded a 4.4% rise in visits from 2014, and total of 11% increment from 2010. Together with the decline in total available ED beds, these factors translate to higher ED crowding. It is important to mention that within the cohort period there have been no significant changes in the workflow or methodologies of admission and discharge either in the ED or in inpatient departments, including staffing levels or financial incentives.

One-week ED readmission rates in 2010, 2014 and 2016 were 6.5%, 6.4% and 6.7% respectively, with *p* = 0.347 for 2010–2014 and *p* = 0.21 for 2010–2016 comparison. These numbers are stable throughout the cohort and are lower than reported by Guttmann et al. In 2011 (one-week revisit rates of 8–14% after ED discharge) [7]. While Gabayan et al. reported lower one-week revisit [16] (2.6%), that cohort was composite of medical and surgical ED, including more than 30% visits due to minor injuries, which lowered the rates of revisits and were not assessed in our cohort.

One-month death rates were stable at around 4%. These rates are comparable and even lower than an Australian study which found that 2, 7 and 30 days mortality was related to both ED and hospital overcrowding, reporting one-month death rates of 5% and 6% for 90–99% occupied and ≥ 100% occupied hospitals [11]. Although Madsen et al. showed linear association between occupancy and death rates in overcrowded hospitals in Denmark, there are several differences: First, our study refers to mortality of patients after ED visits, while the aforementioned study deals with patients who were admitted to hospitalization wards; Second, until recently there have been no EDs in Danish hospitals and patients were admitted directly to the indicated hospital department, in contrast to the Israeli system [12].

Our findings reflect efforts and adaptations made by ED staff, including identifying and prioritizing high risk patients (“cannot stay in the ED” protocol: a protocol which marks a patient as unstable, thus admission to a ward must take place without delay).

Other important changes in the ED were the significant increase in average length of ED visit, average delay time and percentage of patients delayed in the ED after a decision of hospitalization took place. The average visit to the internal ED lasted nearly four and a half hours in 2016, almost 80 min more than the average visit in 2010. Among the patients signed to be hospitalized, mean waiting time in the ED increased from 97 min to four hours in 2016. While in 2010 less than a quarter of the hospitalized patients had to wait in the ED more than 2 h for a bed in hospitalization ward – in 2016 2/3 of admitted patients are being delayed more than 2 h.

These findings, along with decrease in hospitalizations rates in the internal medicine wards reflect change in center of gravity from the hospitalization wards towards the ED, and the state of ED overcrowding. Such overcrowding might take its toll on staff and patients alike – while staff experience burnout and fatigue [15, 17], an increased mortality was documented in overcrowded hospitals and was associated with longer ED duration of stay and longer physician waiting times. [11]

What can potentially be done to alleviate the problematic developments documented in this study?

- The phenomenon of overnight patients being discharged in the morning by senior staff after having been admitted for inpatient care by a resident the previous evening, suggests that the presence of senior emergency physicians during afterhours shifts might decrease such short-lived admissions.
- The creation of short-stay diagnostic units [18] was shown to be beneficial in reducing admissions, by permitting more testing in the ED, senior consults and the initiation of brief therapeutic interventions (e.g. IV antibiotics for 24 h). Such units usually admit patients for less than 24 h and are very efficient use of inpatient beds.
- Improvement in inpatient bed management, such as early morning discharge instead of the almost universal afternoon average discharge time, will make beds available in the inpatient department earlier and shorten boarding of patients in the ED.
- Primary health care centers in the community level should be expanded to funnel unurgent referrals.
- Adjustments in emergency medicine departments size, personnel, medical equipment and imaging techniques should take place to meet the demand.

Our study has several important limitations. First, our findings represent one hospital experience; however, our hospital is one of the largest in Israel and is a national leader in number of patients admitted to internal medicine wards. Out of 296,200 nationwide internal medicine ward admissions in 26 different hospitals in 2015, 23,000 were recorded in the TASMC alone – second only to Soroka Medical Center (23,900) [3]. Our findings correlate with trends presented on the national level, and further providing new data concerning ED discharge and readmission rates.

Second, we analyzed retrospective data which lacks clinical features such as diagnoses and comorbidities, thus reflecting only general trends.

Third, the lack of control group limits the extent to which associations between policy change and outcomes could be assessed; however, as this is a national MOH policy change which affected all the hospitals in Israel, no other relevant control group could be found and analyzed. It is important to mention that a causative relation cannot be made between policy change and the findings of this study, due to the retrospective nature of this work, and because other factors (such as other MOH directives, accreditation requirements, etc.) could also have contributed to changes in ED activity.

Fourth, our data lacks information about socioeconomic and demographic states, which are of great influence on bounce-back and readmission.

The strength of our study relies in its large scale, encompassing more than one hundred and sixty thousand ED visits in a tertiary center, making its findings of statistical, clinical and managemental importance.

## Conclusions

Following the 2011 Ministry of Health’s decision to limit internal medicine wards to 120% occupancy rates, and natural growth in population volume, a significant change was noted in the work of a large, presumably representative emergency department in Israel. In this large scale of real life data, we found an 11% rise in the yearly ED visits, a significant one-fifth reduction in hospital admission rate alongside a significant elevation in ED visit length and rates of admission-delayed patients. However, despite these changes, one-week readmission and one-month mortality rates remained low and unchanged (~ 6.6% and 4.3% respectively). We propose some commonsense interventions which have the potential to alleviate some of the negative findings.

## Abbreviations

ED: Emergency Medicine Department
IQR: Interquartile ranges
LOS: Length of stay
MOH: Ministry of Health
SD: Standard deviations
TASMC: Tel-Aviv Sourasky Medical Center
